# Bullying Experiences, Depression, and the Moderating Role of Resilience Among Adolescents

**DOI:** 10.3389/fpubh.2022.872100

**Published:** 2022-05-25

**Authors:** Li-Yin Lin, Yu-Ning Chien, Yi-Hua Chen, Chih-Yi Wu, Hung-Yi Chiou

**Affiliations:** ^1^Department of Leisure Industry and Health Promotion, National Taipei University of Nursing and Health Sciences, Taipei, Taiwan; ^2^Master Program of Big Data Analysis in Biomedicine, College of Medicine, Fu Jen Catholic University, New Tapei City, Taiwan; ^3^School of Public Health, College of Public Health, Taipei Medical University, Taipei, Taiwan; ^4^Institute of Population Health Sciences, National Health Research Institutes, Miaoli County, Taiwan

**Keywords:** bullying, victimization, depression, resilience, adolescent, mental health

## Abstract

**Introduction:**

Resilience refers to the ability to adapt to difficult situation or adversity. Resilience is what gives people the psychological strength to cope with stress and hardship. Previous studies have investigated the relationship between resilience and bullying victimization and mental health problems. But whether the moderating effect of resilience against depression varies among victims of different types of bullying victimization remains unknown.

**Methods:**

The study used data from the Taiwan Adolescent to Adult Longitudinal Study (TAALS), which was a school based, nationwide, longitudinal study conducted among adolescents in Taiwan. Between 2015 and 2019, the survey was repeated three times to capture changes in health behaviors. Meanwhile, our study is a cross-sectional study focusing on the 2nd follow-up survey of the TAALS, where we recruited 4,771 Grade 7 (12–13 years) and Grade 10 (15–16 years) students who had experienced bullying at school.

**Results:**

This study confirms the protective effect of resilience on depression among adolescents who have experienced bullying. The mode resilience score was used as a reference group. Compared to the reference group, victims of verbal bullying from the lowest resilience group were at the greatest risk of depression (OR = 5.91, CI = 4.38–7.99). Compared to the reference group, victims of cyber bullying from the highest resilience group had the lowest risk of depression (OR = 0.72, CI = 0.57–0.90).

**Conclusion:**

Regardless of the type of bullying victimization, resilience has been shown to offer protection against depression. Specifically, higher resilience levels offer the greatest protection against depression for victims of cyber bullying compared to other three types of bullying victimization. Early interventions to reduce negative effects of bullying victimization may start with increasing an individual's resilience during adolescence.

## Introduction

Adolescence is a critical period of development and the start of the transition to adulthood. One important reason for evaluating young people's experiences of bullying and being victimized by bullies is these experiences have significant associations with a range of mental health problems (1, 2). Being bullied during adolescence is a common, distressing and preventable experience and has been associated with mental illness, substance misuse, and suicide risk in adulthood (3–5). Bullying can take many forms including physical violence, name calling, social exclusion, spreading rumors, and sending insulting or threatening online messages. Exposure to violence has been associated with severe and permanent mental health problems; such as low self-esteem, depression or anxiety, antisocial or disruptive behaviors, academic failure, or self-harm (6–8). Violence here is defined as direct physical abuse, witnessing parental violence, and perceptions of neighborhood violence (6, 8). Studies have suggested that victims of bullying not only have a high risk of developing depression or anxiety but are also likely to commit self-harm (9, 10). Several studies have estimated that approximately 20–35% of adolescents have had at least one experience of bullying, victimization, or both (11, 12). In Taiwan, a recent study estimated that ~19% of junior high school students in Taiwan have experienced violence in school (13). The high prevalence of bullying in schools in Taiwan has prompted calls for Taiwan's government to develop a policy response to prevent bullying in schools.

Although bullying has been strongly correlated with mental health problems among adolescents, some positive psychological traits like “resilience” and “positive-thinking personality” may be protective during stressful circumstances and prevent adolescents from developing mental health problems following a bullying episode (14). Resilience comes from the accumulation of positive interactions with family members, peers, neighbors, and the community, rather than being an inherent personality trait (14, 15). Resiliency theory provides a conceptual framework for understanding why youths grow up to be healthy adults despite exposure to adversity (16–18). Resiliency focuses on positive contextual, social, and individual variables that interfere with the developmental trajectories from adversity to problematic behaviors, mental distress, and poor health outcomes. These positive contextual, social, and individual variables are known as promotive factors, work in opposition to adversity, and help youth to overcome negative effects of adversity exposure (19, 20). The two types of promotive factors identified by Fergus and Zimmerman (19) are assets and resources. Self-efficacy or problem-solving skill are known as positive factors reside within individuals. On the other hand, resources are referred to factors outside individuals such as social support, family support, interpersonal skills, mentorship programs that provide opportunities for youth to learn and practice skills (21). For instance, in a cross-sectional study conducted by Santos et al., it analyzed resilience as a protective factor against the development of depression symptoms and decreased satisfaction with life among victims of cyberbullying (22). In a meta-analysis, resilience was found to correlate negatively with anxiety and depression, but positively with positive indicators of mental health and life satisfaction (23). Adolescents with higher resilience appear to have better outcomes after encountering adversity than adolescents with lower resilience. Resilient individuals bounce back from stressful events more quickly and effectively (24).

Previous studies have investigated the relationship between resilience and bullying victimization and various mental health problems including depression, suicide, or psychiatric disorders. Many studies have shown that adolescents exposed to violence are more likely to have adverse outcomes in adulthood including mental health problems and suicidal thoughts (8, 25). Resilience is likely to be a recovery mechanism that restores individuals' emotional status back to normal after experiencing adversity or stress (26). Other studies have shown that resilience can be strengthened by other protective factors like self-esteem and social support (27, 28).

Many studies have indicated that resilience is negatively related to bullying and cyberbullying, and it moderates the relation between bullying victimization and youths' negative mental health outcomes. For instance, Zhou et al. (29) found resilience is an important factor that mediates the relationship between bullying victimization and childhood depression. Huang and Mossige (30) found that resilience has a significant negative association with poor mental health, and also moderates the negative relation between poly-victimization and young people's mental health. Many recent studies investigating the effect of resilience on mental health have some limitations. For instance, many studies have focused on estimating whether resilience can reduce mental health problems following bullying but few studies have examined if the interaction between resilience, bulling, and mental health problems varies by the type of bullying victimizations (31). Secondly, previous studies have mostly evaluated the mean effect of resilience without considering the range of different resilience levels that bullying victims have. Different levels of resilience may exert different modifying effects on mental illness and psychological distress following bullying. Resilience also showed significant correlation with positive mental health indicators such as life satisfaction and perceived wellbeing (32–34). One study examined late adolescents' resilience as a moderator of the relationship between poly-bullying victimization and subjective wellbeing (35). Therefore, the aim of this study is to examine whether resilience plays a protective role in preventing depression among bullying victims and whether the extent of protection differs depending on the type of bullying victimization and resilience level.

## Methods

### Study Design and Participants

The present study used data from the Taiwan Adolescent to Adult Longitudinal Study (TAALS) (36), which was a school-based, nationally representative, longitudinal study conducted among adolescents between 2015 and 2019. A multistage stratified sampling approach with probability proportional to size sampling was applied to obtain a nationally representative sample of adolescents. The baseline survey for the TAALS was conducted in 2015, and between 2015 and 2019 the survey was repeated three times. During the first wave of the cohort study (Wave 1), 6,903 junior high school students and 11,742 high school students were interviewed, for a total of 18,645 students. Among those 18,645 students, in the second wave of the cohort study (our present study), 4,771 students Grade 7 students (age range: 12–13) and Grade 10 students (age range: 15–16) were identified as ever had bullying experience at school. Bullying experiences were defined as “pure-targets” and “target-perpetrators.” Target-perpetrators refers to participants who were not only bullied but also bullied others. The questionnaire used for the TAALS was developed through a systematic review of multiple large-scale international youth studies. The TAALS was a cohort study funded by Taiwan's Health Promotion Administration (HPA) and our access to the TAALS dataset was granted by the HPA. The original data collection for the TAALS study and our subsequent analysis of the survey results were both approved by the Joint Institutional Review Board of Taipei Medical University, Taiwan (TMU-JIRB-201410043).

### Measurement of Depressive Symptom

Depressive symptoms were evaluated using the Chinese version of the Center for Epidemiological Studies Short Depression Scale (CES-D) (37). Shrout and Yager (38) had examined the sensitivity and specificity of the 5, 10, and 20-item versions of CES-D scale and found the sensitivity and specificity of the 5-item CES-D scale was similar to those of the full 20-item scale. Previous studies have also suggested that researchers can select CES-D items with the highest factor loading through factor analysis. Therefore, we used factor analysis to select 5 items with the 5 highest factor loadings from the 10-item CES-D scale. Depressive symptoms experienced in the past seven days was evaluated using the following questions: (1) I did not feel like eating; my appetite was poor; (2) I could not get “going;” (3) I felt depressed; (4) I felt everything I did was an effort; (5) I felt lonely. The 5-point scale was validated by an internal expert committee meeting who reviewed the validity of different versions of the CES-D scale and selected the final version of the survey. The five-item scale was found to have a high internal consistency with a Cronbach α value of 0.79. Finally, item responses were rated on a four-point Likert scale ranging from 0 to 3 and the sum of all the responses was used to calculate a total score, which ranged from 0 to 15. Participants who obtained a score ≥ 7 were considered to have depression.

### Measurement of Resilience

The questionnaire used to evaluate resilience level was adapted from the Chinese version of the Inventory of Adolescent Resilience (IAR), which is a 28-item questionnaire that was used in a previous study to assess resilience levels among Taiwanese 7 and 9th grade students (39). The IAR contains 4 dimensions: problem solving and cognitive maturity, hope and optimism, empathy and interpersonal interaction, and emotional regulation. In addition to the expert evaluation, like Shrout and Yager (38), we applied factor analysis to select three questions with the highest factor loadings from each of the four dimensions, resulting in 12 questions for the entire survey. The twelve questions used to measure resilience are: “I can solve problems in an organized way,” “I am an optimistic person,” “I can control my emotion when being upset by others,” “I can find effective solutions to the problems, “I am an outgoing person,” “I choose not to react to the people who make fun of me,” “When I'm upset, I usually can quickly return to peace,” “I can make myself happy,” “I can make others feel warm and willing to share emotions and feelings with me,” “I don't give up easily when encountering setbacks,” “I can treat others with kindness and generosity.” These 12 questions were used to measure resilience level and all questions reached a good internal consistency (Cronbach's α coefficient of 0.84). Responses were rated on a 4-point Likert scale ranging from 1 to 4 (strongly disagree, disagree, agree, and strongly agree) and the sum was used to calculate a total score. A higher score indicated that a participant is more resilient.

### Measurement of Bullying Victimization

Participants' bullying experiences and type of bullying victimization were determined in this study. We modified the assessment tool developed by the U.S. Centers for Disease Control and Prevention (CDC) called “Measuring Bullying Victimization, Perpetration, and Bystander Experiences” (40) to determine whether participants had been exposed to bullying at school during the past 6 months. We used the following four questions to assess participants' bullying experience during the past 6 months: “I was pushed, shoved, slapped, or kicked by other students,” “I was teased by other students,” “I was ignored or felt left out of activities or games on purpose,” and “Some pictures or words were posted online, (through email, computer text message, or Facebook), by someone else to make others laugh.” Each question was rated on a 5-point scale ranging from 0 being “Never” to 4 being “Always.” If students scored 1–4 for one of the questions, they were identified as victims. In addition, we adopted a similar approach to identify students' bullying victimization type. For example, if participants scored 1–4 on the question “I was pushed, shoved, slapped, or kicked by other students,” they were identified as being a victim of physical bullying. If participants scored 1–4 on the question “I was teased by other students,” they were identified as being a victim of verbal bullying. If participants scored 1–4 on the question “I was ignored or felt left out of activities or games on purpose,” they were identified as being a victim of relational bullying. Finally, if participants scored 1–4 on the question “Some pictures or words were posted online by someone else to make others laugh,” they were identified as being a victim of cyber bullying.

### Measurement of Social and Family Support

The level of peer support was quantified using a 5-item questionnaire developed by the U.S. Centers for Disease Control and Prevention (CDC) (40). Questions used to access peer support during the past 6 months included “My classmates/friends truly care about things that happened to me,” “When I am in need for help, my classmates/friends will help me,” “I have classmates/friends that I can trust,” “My classmates/friends care about my feelings,” “My classmates/friends only care about themselves,” and “My classmates/friends think that I'm not good enough.” Responses were rated on a 4-point scale ranging from 0 (None of them) to 3 (All of them) and summed up to calculate the total score. The total score ranged from 0 to 15, and the higher score indicated stronger peer support. The level of perceived support from family was measured by the 6-item questionnaire adapted from the 40-item Inventory of Socially Supportive Behaviors (ISSB) (41). The questions used to access family support during the past 3 months included: “My family members understand and support my decisions and behaviors,” “My family members are willing to listen when I need to vent about something,” “When I'm feeling down, my family members will talk to me and encourage me,” “My family members will fully support me regardless the cost when is necessary,” “When I encounter some problems, my family members will share their solutions with me,” and “When I need to make a decision, my family members will discuss and share their ideas with me.” Responses were rated on a 4-point scale ranging from 0 (Never) to 3 (Always) and summed up to calculate the total score. The total score ranged from 0 to 18, and the higher score represented more family support received.

### Confounders

Sex, academic grade level, family support, and peer support were measured at baseline and were incorporated as potential confounders in both the regression and the RCS models. Additionally, socioeconomic factors including mother's highest level of education achieved (junior high school graduate or below, senior high school graduate, or University graduate), mother's ethnicity (Chinese/Aboriginal/immigrant), father's employment status (full-time, part-time, unemployed), and household structure (live with parents and grandparents, live with parents, live with only grandparents, live with collateral relatives) are also important confounders affecting an individual's resilience level, and were adjusted in both the regression and the RCS models.

### Statistical Analysis

**Figure 1:**
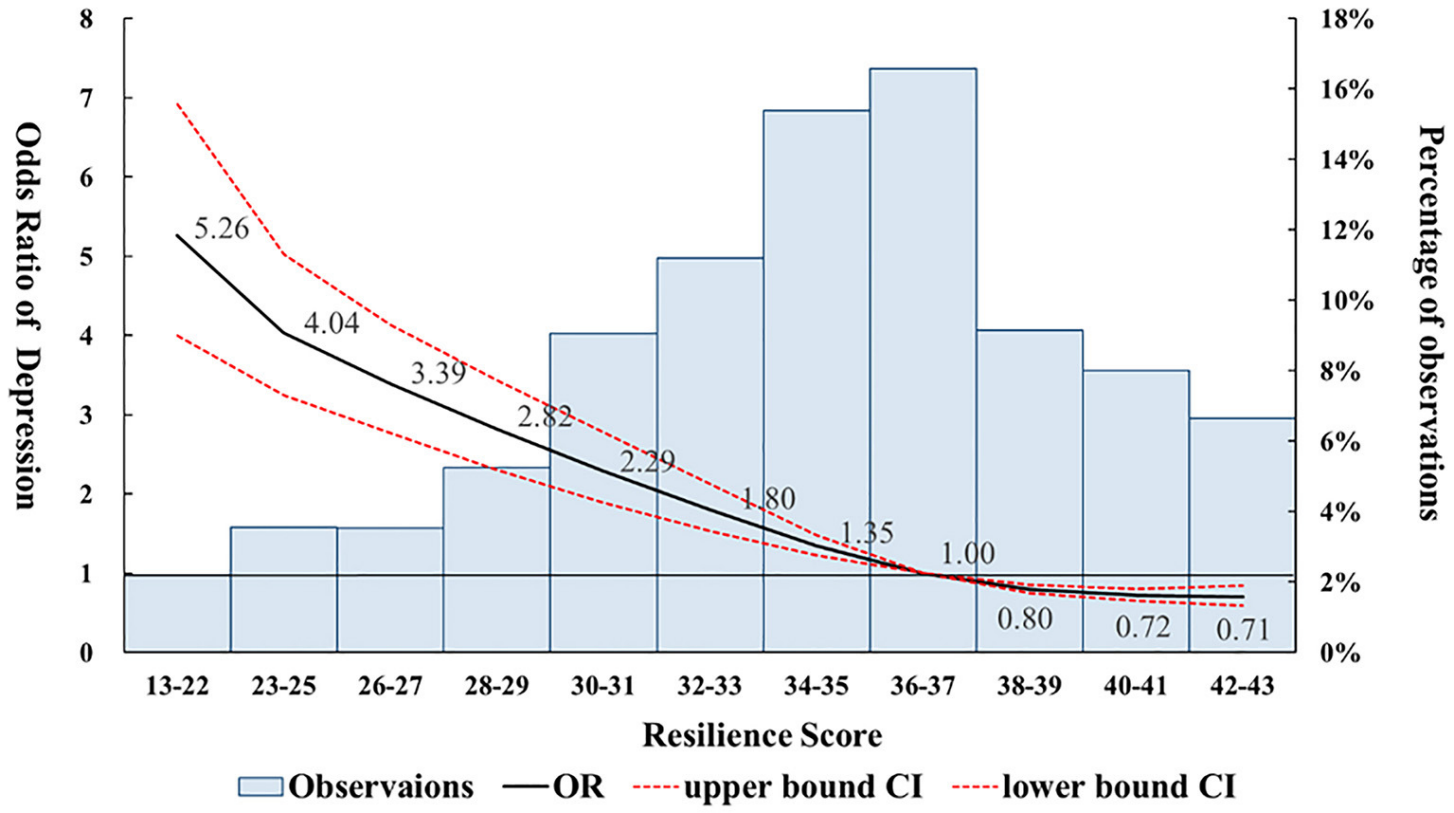
Odds ratios of depression stratified by different resilience scores among victims of bullying. The odds ratios were calculated using a Restricted Cubic Spline (RSC) regression model, adjusted for gender, age, peer support, family support, mother's ethnicity, mother's education, father's employment status, and household structure. The reference group was defined at the 50^th^ percentile resilience level, which included all the resilience scores from 36 to 37.

**Figure 2:**
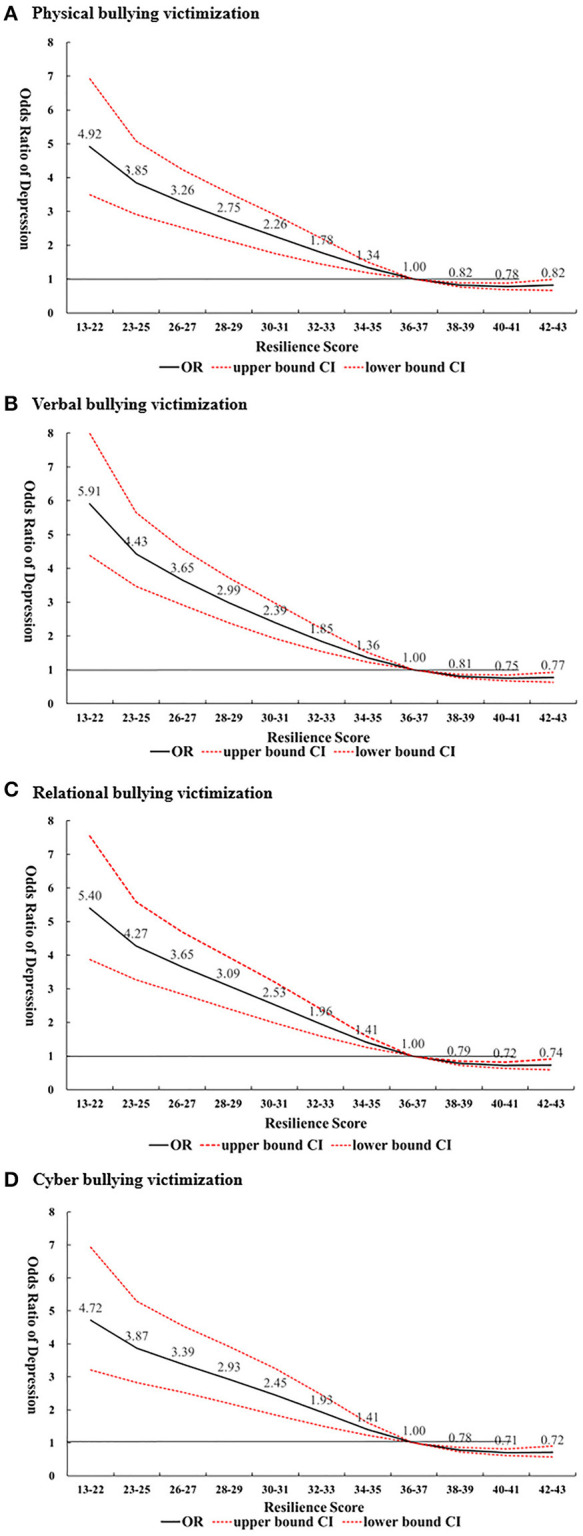
Odds ratio of depression stratified by resilience score and type of bullying victimization. **(A)** Physical bullying victimization. **(B)** Verbal bullying victimization. **(C)** Relational bullying victimization. **(D)** Cyber bullying Victimization. The odds ratios were calculated by the Restrict Cubit Spline (RCS) Regression model, adjusted by gender, age, peer support, family support, mother's ethnicity, mother's education, father's employment status, and household structure. Reference group was defined at the 50^th^ percentile resilience level, which included all the resilience scores from 36 to 37.

Descriptive statistics were used to describe individual and socioeconomic characteristics among participants with and without depression. Differences in bullying experience (pure targets/target-perpetrators), bullying victimization type, resilience level, mother's ethnicity, mother's education level, father's employment status, household structure were evaluated using a chi-square test. Because there are currently no universal cutoff values available to define levels of resilience, we therefore took an approach of dividing the resilience scores into six groups according to centiles. The six levels of resilience were classified as the following: ≤ 10th percentile (resilience score ≤ 28), 11–20th percentile (resilience scores between 29 and 31), 21–40th percentile (resilience scores between 32 and 34), 41–60th percentile (resilience scores between 35 and 37), 61–80th percentile (resilience scores between 38 and 41), and ≥80th percentile (resilience scores ≥ 42). In order to capture any change in the protective effect from different levels of resilience against depression, two methods were used: a logistic regression model and a restricted cubic spline (RCS) regression model (42). First, we divided resilience levels into six categories by percentile and performed a logistic regression to evaluate the odds ratio of depression among all participants being bullied at school (**Table 2**). Next, we performed another logistic regression to evaluate the association between resilience level and depression stratified by bullying victimization type (i.e., physical, verbal, relational, and cyber bullying victimization) (**Table 3**). Since logistic regression models are unable to capture the continuous changes of non-linear factor (i.e., resilience), we performed a restricted cubic spline (RCS) regression to examine the association between resilience and depression among bullied participants (Figure 1). Next, the association between resilience level and depression stratified by bullying victimization types was also analyzed by the RCS regression (Figure 2). All statistical analyses were conducted using STATA 12 statistical software and results were considered significant at *p* < 0.05.

## Results

### Participants Baseline Characteristics

Participants' characteristics at baseline are presented in Table 1. Among the 4,771 participants there were slightly more participants from Grade 10 than from Grade 7 students (53 vs.47%), and more male than female participants (53 vs. 48%). There were more target-perpetrators than pure targets (71 vs. 29%) and verbal bullying victimization was most common among the four types of bullying victimization (77.2%). Most of the participants had a resilience level at 21–40th and 61–80th percentiles (21.4 and 20.8%), most lived with their parents (75%), and had their father working full-time (83%). Among all the variables, sex, type of bullying victimization, resilience level, father's employment status, household structure, and social support (i.e., peer and family support) were significantly different in participants with depression compared to those without depression. The results showed no significant difference in bullying experience, mother's ethnicity, and mother's education level among participants with depression compared to participants without depression.

**Table 1:** Baseline characteristics among victims of bullying with and without depression.

**Variables**	**Total (*****N*** **=** **4,771)**		**With depression (*****N*** **=** **938)**		**Without depression (*****N*** **=** **3,833)**		***P*-value**
	***n***	***%***	***n***	***%***	***n***	***%***	
**Grade level of students**							0.826
Grade 7	2,243	47.01	444	47.33	1,799	46.93	
Grade 10	2,528	52.99	494	52.67	2,034	53.07	
**Gender**							**0.007**
Male	2,497	52.34	454	48.40	2,043	53.30	
Female	2,274	47.66	484	51.60	1,790	46.70	
**Bullying experience**							0.207
Pure targets	1,382	28.97	256	27.29	1,126	29.00	
Target-perpetrators	3,389	71.03	682	72.71	2,707	69.71	
**Victimization type**							
Physical	2,755	57.74	593	63.22	2,162	55.68	**<0.001**
Verbal	3,684	77.22	774	82.52	2,910	74.94	**<0.001**
Relational	2,958	62.00	640	68.23	2,318	59.70	**<0.001**
Cyber	1,979	41.48	452	48.19	1,527	39.33	**<0.001**
**Resilience level**							**<0.001**
≤ 10 percentile	706	14.80	286	30.49	420	10.82	
11–20 percentile	678	14.21	193	20.58	485	12.49	
21–40 percentile	1,022	21.42	184	19.62	838	21.58	
41–60 percentile	791	16.58	111	11.83	680	17.51	
61–80 percentile	994	20.83	112	11.94	882	22.71	
≥80 percentile	580	12.16	52	5.54	528	13.60	
**Mother's ethnicity**							**0.004**
Chinese	4,082	85.56	781	83.26	3,301	85.01	
Aboriginal	177	3.71	30	3.20	147	3.79	
Immigrant	430	9.01	101	10.77	329	8.47	
Unknow	82	1.72	26	2.77	56	1.46
**Mother's education**							**0.002**
Junior high school graduate or below	723	15.15	152	16.20	571	14.71	
Senior/vocational high school graduate	1,998	41.88	375	39.98	1,623	41.80	
University graduate	1,614	33.83	297	31.66	1,317	33.92	
Unknow	436	9.14	114	12.15	322	8.40
**Father's employment status**							**<0.001**
Full-time	3,968	83.17	728	77.61	3,240	83.44	
Part-time	195	4.09	51	5.44	144	3.71	
Unemployment	546	11.44	141	15.03	405	10.43	
Unknow	62	1.30	18	1.92	44	1.15
**Members in the household**							**<0.001**
Parents	3,573	74.89	648	69.08	2,925	75.33	
Single parent	915	19.18	219	23.35	696	17.92	
Grandparents	148	3.10	29	3.09	119	3.06	
Collateral members	135	2.83	42	4.48	93	2.40	
	**Mean**	**SD**	**Mean**	**SD**	**Mean**	**SD**	***P*** **-value**
Peer Support	13.19	2.17	12.67	2.19	13.32	2.15	**<0.001**
Family Support	15.00	4.80	13.64	4.81	15.33	4.75	**<0.001**

***p < 0.001, **p < 0.01, *p < 0.05. The bold values are referring to those with statistical significance.

### Association Between Resilience Level and Depression

For our regression models, we used the 41–60th percentile resilience level group as the reference group. In the crude model, lower resilience levels, (i.e., ≤ 10th percentile, 11–20th percentile, and 21–40th percentile), were significantly associated with an increased risk of depression when compared to the reference group. In addition, higher resilience levels, (i.e., 61–80th percentile and ≥80th percentile), were associated with a reduced risk of depression when compared to the reference group, however the results were not significant. In the final adjusted model, (adjusting for grade level, age, peer support, family support, mother's ethnicity, mother education, father's employment status, and household structure), similar findings were observed where lower resilience levels were significantly associated with increased risk of depression compared to the reference group. Higher resilience levels were associated with a reduced risk of depression compared to the reference group although the association was not significant (Table 2).

**Table 2:** Odds ratio of depression stratified by different resilience levels among victims of bullying.

	**Dependent variable: depression**			
	**Crude model**		**Adjusted model**	
	**OR**	**95% CI**	**OR**	**95% CI**
Resilience levels				
≤ 10th percentile	4.17***	(3.25–5.36)	4.07***	(3.07–5.38)
11–20th percentile	2.44***	(1.88–3.16)	2.51***	(1.87–3.35)
21–40th percentile	1.35**	(1.04–1.74)	1.47***	(1.11–1.95)
41–60th percentile	Reference	N/A	Reference	N/A
61–80th percentile	0.78	(0.59–1.03)	0.95	(0.70–1.29)
≥80th percentile	0.60***	(0.43–0.85)	0.73	(0.50–1.07)

The crude model was a simple logistic regression. The adjusted model was adjusted for gender, age, peer support, family support, mother's ethnicity, mother's education, father's employment status, and household structure. Robust standard deviation was used. A trend test was also completed to examine if resilience score (from low to high) as a continuous variable, has a trend effect on depression and the effect was found to be significant (p-trend = < 0.0001). ***p < 0.001, **p < 0.01, *p < 0.05.

### Association Between Resilience Level and Depression Stratified by Bullying Victimization Type

We investigated whether the type of victimization plays a role in the association between resilience level and depression. Among all four types of bullying victimization, as the resilience level increased, the risk of depression significantly decreased when compared to the reference group (i.e., 41–60th percentile). Additionally, for all types of bullying victimization, the highest resilience level (≥80 percentile) was associated with a reduced risk of depression compared to the reference group, although the association was not significant (Table 3).

**Table 3:** Odds ratio of depression stratified by resilience level and type of bullying victimization among victims of bullying.

	**Dependent variable: Depression**			
	**Model 1: Physical bullying victimization (*n* = 2,455)**	**Model 2:** **Verbal bullying victimization** **(*n* = 3,319)**	**Model 3: Relational bullying victimization (*n* = 2,654)**	**Model 4:** **Cyber bullying victimization** **(*n* = 1,777)**
Resilience levels				
≤ 10th percentile	3.72*** (2.60–5.31)	4.17*** (3.06–5.68)	4.53*** (3.19–6.44)	4.04*** (2.69–6.07)
11–20th percentile	2.60*** (1.80–3.77)	2.35*** (1.70–3.25)	3.26*** (2.28–4.64)	2.68*** (1.75–4.10)
21–40th percentile	1.31 (0.91–1.88)	1.43*** (1.05–1.96)	1.56** (1.09–2.23)	1.50* (0.99–2.27)
41–60th percentile	Reference	Reference	Reference	Reference
61–80th percentile	1.02 (0.69–1.50)	0.86 (0.61–1.22)	1.07 (0.73–1.56)	1.03 (0.66–1.60)
≥80th percentile	0.84 (0.53–1.32)	0.80 (0.53–1.21)	0.84 (0.52–1.36)	0.76 (0.45–1.26)

Each model was adjusted for gender, age, peer support, family support, mother's ethnicity, mother's education, father's employment status, and household structure. Robust standard deviation was used. A trend test was also performed to examine if resilience score (from low to high) as a continuous variable, has a trend effect on depression. The trend effects in all four models were found to be significant (p = < 0.0001). ***p < 0.001, **p < 0.01, *p < 0.05.

### Odds Ratio of Depression Calculated by Restricted Cubic Spline Regression

The logistic model showed that lower resilience levels were significantly associated with a higher risk of depression when compared to the reference group. However, the results did not show higher resilience levels were significantly associated with a lower risk of depression using a linear logistic model. Since the relationship between resilience and depression may not always be linear, the RCS model was used to examine the association between these two variables. The RCS model demonstrated that higher resilience levels are significantly associated with a lower risk of depression among victims of bullying. Figure 1 shows the odds ratio of depression among participants who were bullied stratified by resilience score. The resilience score at the mode (scores between 36 and 37) was used as reference group to reflect most commonly occurring resilience level among participants. Overall, our results revealed an inverse, non-linear association between higher resilience scores and the risk of depression among bullied participants. Participants who experienced bullying and had a resilience level score of 38 or above had a significantly reduced risk of depression when compared to the reference group. In contrast, participants who experienced bullying and had a resilience level score of 35 or lower had a significantly increased risk of depression when compared to the reference group.

### Odds Ratio of Depression at Different Resilience Levels Stratified by Different Types of Victimization

In Figure 2, we further stratified the participants based on the type of bullying victimization, (i.e., physical, verbal, relational, and cyber victimization), to investigate whether the type of bullying victimization modifies the protective effect that resilience has against depression. Our results showed that for the lowest resilience level group (score 13–22), victims of verbal bullying had the highest risk of depression (OR = 5.91; CI:4.38–7.99), followed by victims of relational bullying (OR = 5.40; CI: 3.87–7.55), victims of physical bullying (OR = 4.92; CI: 3.49–6.92), and victims of cyber bullying (OR = 4.72; CI: 3.21–6.93) compared to at the reference resilience level group for each type of bullying (score 36–37). Furthermore, for the highest resilience group (score 42–43), victims of cyber bullying had the lowest risk of depression (OR = 0.72; CI: 0.57–0.90), followed by victims of relational bullying (OR = 0.74; CI: 0.60–0.91), victims of verbal bullying (OR = 0.77; CI: 0.64–0.93), and victims of physical bullying (OR = 0.82; CI: 0.67–1.00) compared to those from the reference resilience level group for each type of bullying (score 36–37).

## Discussion

This study not only confirms the protective effect resilience has against depression among young adolescents who have experienced bullying; to the best of our knowledge, it is also one of very few studies to investigate whether different types of bullying victimization affect the strength of the protective effect that resilience has on depression. Our results showed that a lower resilience score was significantly associated with a higher risk of depression in both the logistic regression model as well as the RCS model. However, higher resilience scores were significantly associated with a reduced risk of depression only in the RCS model. Furthermore, the highest resilience level offers the greatest protection against depression for victims of cyber bullying compared to victims of other types of bullying victimization. In contrast, the lowest resilience level was associated with the highest risk of developing depression in victims of verbal bullying compared to victims of other types of bullying victimization.

Consistent with previous studies (43, 44), our main findings indicate that a lower resilience score is associated with an increased risk of depression, while a higher resilience score appears to be protective for victims of bullying against depression. When we first performed the logistic regression to analyze the association between resilience and depression among adolescents who have experienced bullying, our results showed low resilience levels were significantly associated with an increased risk of depression. High resilience levels, on the other hand, were associated with a decreased risk of depression although the association was not significant. When we further performed a restricted cubic spline (RCS) regression, we observed an inverse, non-linear association between resilience level and risk of depression among adolescents with bullying experience. This inverse, non-linear association was consistent with other studies' findings: adolescents who have experienced bullying, (both pure targets and target-perpetrators), usually have a higher risk of depression and suicidal thoughts (45, 46). However, resilience appears to reduce the risk of depression among adolescents who have experienced bullying (15). The consequences of bullying can be severe and long-lasting and include: lower self-esteem, academic failure, behavior problems, psychosis and feelings of hopelessness (47). Resilience often refers to the process of adapting well in the face of significant adversity or stress (16). In many empirical studies, resilience is found to be inversely correlated with indicators of mental illness such as depression, anxiety, and negative emotions, and positively associated with positive indicators of mental wellness, such as subjective wellbeing and positive emotions (23).

Unlike previous studies that examined bullying in general, our present study studied by type of bullying victimization and examined if different types of bully victimization were associated with different risks of depression. Our results indicated that victims of verbal bullying were at the highest risk of depression, followed by victims of relational bullying, victims of physical bullying, and victims of cyber bullying when the resilience level was below the reference group. This finding was consistent with another similar study examining bullying victimization and adolescent mental health, where they also found individuals reporting more frequent verbal bullying experienced higher levels of depression (48). According to our findings, resilience appears to provide varying levels of protection based on the type of bullying victimization. Among all four types of bullying victimization, our results showed higher resilience levels, (above reference group), were associated with greater protection against depression for victims of cyber bullying, followed by relational bullying, verbal bullying, and physical bullying. Despite a large number of studies focusing on the relationship between bullying and depression, very few have examined the associations between the type of bullying victimization, resilience, and depression. Additionally, many previous studies have focused primarily on general bullying, (all kinds of bullying experiences), rather than examining each type of bullying victimization separately. Future research is needed to explore why the modifying effect that resilience has on the association between bullying and depression is different based on the types of bullying victimization (i.e., physical/verbal/relational/cyber bullying).

Because our study provides further evidence of the inverse association between resilience level and depression, it is worthwhile to identify the variables that may enhance resilience. A study of young adult American college students, (aged between 18 and 24), concluded that resilient functioning can be improved by individual and environmental protective factors like emotional intelligence (EI), spirituality, and social support (43). EI is comprised of qualities such as understanding one's feelings, differentiating between emotions, and recognizing the influence that one's emotions may have on others. Such abilities can be trained and enhanced with many cognitive and behavioral therapeutic techniques (49). Several other studies also have shown that various forms of spirituality, are linked to enhanced resilience (50). In a review of research on adolescent spirituality and mental health, Wong et al. (51) found that most studies showed a positive relationship between spirituality and adolescent mental health. Another protective factor associated with resilience is social support. Social support is a key correlate of psychological resilience, and preclinical and clinical research finds that weak social support and isolation are associated with indicators of compromised physical and mental health (52). Social support often refers to support received from parents and peers. Young people who have a good, supportive relationship with their parents are able to build supportive relationships with friends, which in turn is associated with better psychological wellbeing (53). However, it has been argued that during adolescence, individuals start spending more unsupervised time with their peers and friends and begin relying upon them more than parents for support (54). Maintaining positive peer relationships has been shown to be associated with a lower risk of being bullied and limit the severity of any bullying that does occur (55). In summary, social support plays an essential role in promoting positive outcomes for students who have experienced bullying during adolescence.

### Clinical and Policy Implications

The findings from our study have several important clinical and policy implications. First, our results suggest that during adolescence clinical interventions should focus on individuals who seem the most isolated, because low social support was associated with lower resilience and a higher risk of depression. Second, improving resilience level may help prevent mental health problems among students who have experienced bullying. This suggests that intervention programs to enhance resilience levels among adolescents could reduce the risk of depression among adolescents. Since resilience can be enhanced by social support, school administrators could develop educational materials for teachers and academic counselors about the signs of disengagement and what steps can be taken to connect students with support networks (43). In addition, schools could offer resilience training by introducing classes that focus on teaching students resilience skills such as: positive reinterpretation, humor, active coping, planning and handling problems, seeking help and social support (56). Next, because higher emotional intelligence (EI) is associated with higher resilience, schools could introduce learning lessons related to cognitive and behavioral change techniques that have been shown to increase EI among students (57). Finally, since some of the participants enrolled in our study are considered young adolescents (those who were 12–13 years old), they may not be aware of the signs of depression. Young adolescents may benefit from psychoeducational materials that include specific information regarding the signs and symptoms of depression that require medical attention, as well as strategies to improve resilient functioning.

### Limitations and Strengths

The main strengths of the study lie in its use of a representative sample of adolescents and the use of standardized and previously validated measures of depressive symptoms. Furthermore, to the best of our knowledge, our study is also the first study to investigate the interaction among different types of bullying victimization, the risk of depression, and levels of resilience. We found that high resilience is protective against depression, regardless of the type of bullying victimization, and this finding has significant clinical and policy implications.

A primary study limitation here is the lack of consistent conceptualization and unified methodology around the definition and utility of resilience measures (58). This has hindered comparisons of findings and conclusions from resilience in adolescence research. Therefore, our study could only use the mode of resilience level among our study participants as the reference group in order to estimate the effect of resilience against depression in different resilience level groups, (i.e., resilience level below or above the reference group). Next, we used the cross-sectional data from the TAALS, resulting in a smaller sample size that may not accurately reflect the bullying situation and presence of depression among all adolescents in Taiwan. Additionally, the cross-sectional study design precludes the possibility of investigating potential variability in resilience over time and makes causal inference difficult. Resilience may be more or less protective against depression depending on what other factors are present (i.e., social support, self-esteem, emotional intelligence, etc.). Therefore, further intervention research is needed to clarify the causal relationships between bullying, resilience and depression. Finally, the use of self-reported data may introduce response bias, as some participants may not feel comfortable providing accurate responses to some sensitive items, such as the frequency of bullying experienced at school or the presence of depressive symptoms.

## Conclusion

Our study results demonstrate that victims of bullying are at higher risk for depression and that resilience plays an important protective modifying role in the association between bullying victimization and risk of depression. Among the types of bullying victimization examined in this study, higher resilience levels offer the greatest protection against depression for victims of cyber bullying. On the other hand, lower resilience levels are associated with the highest risk of depression for victims of verbal bullying. Therefore, strengthening resilience levels among adolescents is central to promoting long-term positive mental health outcomes. Likewise, efforts to counsel youth experiencing bullying victimization may improve if the type of victimization (i.e., verbal, physical, relational, or cyber) are taken into consideration.

## Data Availability Statement

Data are available from the authors upon reasonable request and with permission of the Taiwan Health Promotion Administration. Requests to access the datasets should be directed to H-YC, hychiou@tmu.edu.tw.

## Ethics Statement

The studies involving human participants were reviewed and approved by Taipei Medical University. Written informed consent to participate in this study was provided by the participants' legal guardian/next of kin.

## Author Contributions

L-YL, Y-NC, and H-YC: conceptualization. Y-NC, Y-HC, and L-YL: methodology. Y-NC, Y-HC, and H-YC: validation. Y-NC: formal analysis. L-YL and Y-NC: investigation, writing—original draft preparation, and writing—review and editing. C-YW and H-YC: data curation. Y-HC and H-YC: supervision. All authors have read and agreed to the published version of the manuscript.

## Funding

The work was supported by the Taiwan Health Promotion Administration (HPA), Ministry of Health and Welfare (Grant Number: MOHW105-HPA-H-114-133708).

## Conflict of Interest

The authors declare that the research was conducted in the absence of any commercial or financial relationships that could be construed as a potential conflict of interest.

## Publisher's Note

All claims expressed in this article are solely those of the authors and do not necessarily represent those of their affiliated organizations, or those of the publisher, the editors and the reviewers. Any product that may be evaluated in this article, or claim that may be made by its manufacturer, is not guaranteed or endorsed by the publisher.
